# Continuous work-related sitting time and its association with perceived workplace support for health among workers in the Greater Accra Municipality: a cross-sectional analysis with sensitivity analyses

**DOI:** 10.1186/s12889-024-20572-z

**Published:** 2024-11-05

**Authors:** Emelia Danquah, Nestor Asiamah

**Affiliations:** 1 Department of Procurement and Supply Science, Koforidua Technical University, Eastern Region, Koforidua, Ghana; 2 Division of Interdisciplinary Research and Practice, School of Health and Social Care, University of Essex, Wivenhoe Park, Colchester, CO4 3SQ UK; 3 Department of Health Promotion, Africa Centre for Epidemiology, Accra, Ghana, P. O. Box AN 16284, Accra North, Ghana; 4 International Public Health Management Programme, University of Europe for Applied Sciences, Reiterweg 26B, 58636 Iserlohn, Germany; 5 Research Faculty, Berlin School of Business and Innovation, 97-99 Karl Marx Strasse. 12043, Berlin, Germany

**Keywords:** Sitting time, Sedentary behaviour, Workplace support for health, Ghana

## Abstract

**Background:**

Research to date has shown that work-related sitting time can be a major occupational health risk. This understanding has encouraged several workplace health promotion efforts. Even so, some domains of work-related sitting time and their associations with Perceived Workplace Support for Health (PWSH) have not been considered in research. This study aims to compare domains of work-related sitting time between employee characteristics (e.g., gender and age) and assess their associations with PWSH.

**Methods:**

This study adopted a cross-sectional design with a sensitivity analysis against confounding and measures against common methods bias. The study population was employees of private and public firms in Accra, Ghana. In all, 1000 employees participated in the study. The hierarchical linear regression analysis and the independent samples *t*-test were used to present the results.

**Results:**

Employees working in service firms, compared with those working in manufacturing firms, reported longer sitting time for lunchtime. After adjusting for physical function, we found a negative association between PWSH and the sum of continuous sitting time as well as its domains (p < 0.001), except for ‘sitting with a desk’ (p > 0.05).

**Conclusion:**

Work-related sitting time was associated with employee characteristics and was lower at higher PWSH. This study reinforces the importance of workplace support for health and its role in work-related sitting.

**Supplementary Information:**

The online version contains supplementary material available at 10.1186/s12889-024-20572-z.

## Introduction

Work-related sitting is not only an occupational health risk but is also a threat to employee and organizational performance [1, 2]. Work-related sitting time is the amount of time spent by employees sitting to perform a job task [3, 4]. We operationally define work-related sitting time as the amount of time spent on a typical day by an employee sitting at work or in the process of travelling to and from work. This extended definition is premised around our idea that occupational sitting does not only occur at work but also occurs during travel in a car to and from work. Moreover, sitting necessitated by one’s presence at work [e.g., sitting while eating or chatting with workmates during lunchbreak] can be ideally classified as work-related sitting. Research utilising subjective measures, nevertheless, has focused on work-related sitting around the employee’s work desk with or without a screen [e.g., a computer]. We refer to this traditional aspect of work-related sitting time as *sitting with a desk*.

Also of interest are four other aspects or domains of work-related sitting time implied by the above operational definition, namely sitting in a car while travelling to work, sitting in a car while travelling back from work, sitting at meetings, and sitting while eating or when chatting with friends during lunchtime. We have observed from the extant literature [5–7] that these four domains of work-related sitting time have not been sufficiently evaluated in research.

Furthermore, employee performance [8, 9] and health indicators [2, 10, 11] are negatively associated with work-related sitting time. The risk of chronic disease (e.g., cancer and heart disease)  increases with increasing sitting time [1, 8, 12]. These risks, including occupational stress and burnout, are associated with poor job performance and satisfaction [10]. As such, interventions buffering the above work-related sitting times are a necessary way to maximise employee health and productivity.

While many studies have assessed the effect of specialized or purpose-specific workplace health promotion interventions on work-related sitting time [1, 5, 13, 14], a systematic review [10] and our review of recent research suggest that there is no identifiable study evaluating the association between work-related sitting time and Perceived Workplace Support for Health (PWSH), a new construct regarding basic measures in the organization to support employee health [15]. PWSH is a facet of workplace health promotion that enables employees to act and make decisions in favour of their health. It comprises the presence of health champions who encourage healthy habits and availability of support for avoiding an unhealthy lifestyle [15]. It includes availability of occupational policies that guide workplace health promotion. As such, PWSH can encourage health-seeking behaviours (e.g., physical activity) or discourage excessive workplace sitting, which implies its negative association with occupational sitting. PWSH is based on the Salutogenesis Model [16, 17], which emphasises factors that protect health rather than those causing disease. It assumes that empowering people to act and make decisions in favour of their health can result in reduced health risks and improved health.

Therefore, this study aims to, for the first time, examine the associations between PWSH and work-related sitting time measured in terms of the foregoing five domains. This study, thus, builds upon a recent study [15] that developed a short scale for measuring PWSH. This scale suggests that workplace support for employees’ healthy habits and the availability of employees serving as health champions as well as basic health education activities for healthy lifestyles are the core attributes of PWSH. They are a primary way every organization can maintain or improve occupational health [15]. This study investigates whether these basic attributes of PWSH can be associated with work-related sitting time.

The extant literature suggests that physical activity differs across the employee’s industry [i.e., service vs. manufacturing firms] and sector (i.e., public vs. private firms) of employment as well as job type (i.e., full-time vs. part-time) [18, 19]. More specifically, some studies [18, 20, 21] suggest that sitting time may be higher among employees working in service firms, compared with employees of manufacturing organizations. Hence, work-related sitting time is compared between these two groups. Comparisons between full-time and part-time works and between public and private sector workers are also performed. The evidence from these comparisons can unfold groups in a higher need of workplace support for health. This comparison is an extension of evidence from some related studies [6, 22] and is expected to guide the design of potential prospective studies.

Also worth mentioning is the idea that work-related sitting cannot be avoided since employees need to sit for some time to get to work and accomplish some job tasks, but experts have acknowledged a need for employees to avoid prolonged (continuous) sitting of more than 30 min [23]. We operationally define continuous work-related sitting time as the employee’s longest sitting on a typical day that is uninterrupted by a walk or any other physical activity. Sitting time is more harmful when it is continuous and long [23], but previous studies have measured workplace sitting by asking respondents to report broken or interrupted episodes of sitting [8, 12, 22]. Thus, studies focused on continuous sitting time is needed to diversify the literature and to emphasise a need for employees to avoid long episodes of sitting.

This study, therefore, attempted to answer the following research questions: *(1) is continuous work-related sitting time associated with employees’ job type as well as industry and sector of employment*, and *(2) is work-related sitting time associated with PWSH?* Sitting time was measured in five domains: sitting to and from work, and sitting during lunch, meetings, and while working around a desk. This study is expected to provide evidence for a potential prospective study assessing the effect of PWSH on the five domains of continuous work-related sitting time. It is also expected to identify employee groups (e.g., men vs. women) in a higher need of workplace support for health and to produce implications for workplace health promotion. An assessment of the foregoing association would provide insight into the probability of workplace support for health predicting low sitting time. This study would provide vital information for the design of larger population-based surveys. Effect sizes and other statistics from this study can be used for sample size calculation in larger cross-sectional, quasi-experimental, and experimental studies.

## Materials and methods

### Design

This study adopted a cross-sectional design, which involved the use of a self-reported questionnaire and Hierarchical Linear Regression (HLR) analysis to analyse the data.

### Population, sample, and selection

The study setting was Greater Accra Municipality, specifically areas (i.e., Ga Central Municipality and Ga West Municipality) where a cluster of organizations and their employees could be accessed. The participants lived in these areas or in other suburbs of Accra. We selected participants by first identifying 29 firms [i.e., service firms = 19; manufacturing firms = 10] that had and were implementing basic workplace health promotion policies. The manufacturing firms were producers of food, cement, and other consumer products. Generally, employees in such companies work in factories, warehouses, and workshops where physical activity is frequently performed. The service companies were banks, insurance service providers, telecommunication service producers, and agencies of government. Employees in these companies typically work in an office environment as managers, customer care personnel, cashiers, or secretaries. We interviewed personnel managers to ensure these organizations were rolling out workplace health promotion programmes.

We employed purposive sampling to select participants who could provide data for answering our research questions. The inclusion criteria used are: (1) having been a permanent resident of Accra; (2) having a minimum of a basic educational qualification (i.e., basic school leaving certificate), which we used as an indicator of the ability to complete questionnaires in English; (3) the ability to walk for at least 10 min unaided, and (4) willingness to participate in the study voluntarily. Some employees, especially those who are older, may be frail or physically challenged. Such employees may spend more time sitting owing to their physical condition. With the third inclusion criteria, we made sure every participant had the potential to walk or avoid excessive sitting. The purpose of this study would be defeated if employees could not avoid sitting owing to conditions beyond their control. The study was focused on permanent residents of Accra to avoid potential outliers from residents from neighbouring towns such as Nsawam and Kpong who commuted to Accra occasionally for work purposes. Such employees work in Accra but do not permanently live in Accra.

Personnel managers of the organizations provided a database of employees who agreed to participate in the study and consented for their information to be shared with us. The database contained emails and phone numbers. Two research assistants used a questionnaire to screen potential participants and select eligible ones over three weeks via a phone call. A total of 1,292 employees met the inclusion criteria. We calculated the minimum sample required for the study with the G*Power 3.1.9.4 program using relevant statistics [i.e., effect size = 0.2; power = 0.8; α 0.05]. The minimum sample size reached for hierarchical linear regression analysis with a maximum of 19 predictors included in this study was 119. To maximise the response rate, we tried to collect data on all eligible participants.

### Variables and the questionnaire

PWSH was measured using a standardised 5-item scale associated with five descriptive anchors (i.e., strongly disagree – 1, disagree – 2, somewhat agree – 3, agree – 4, and strongly agree – 5). This scale produced a Cronbach α = 0.82 in a previous study [15] from which it was adopted and a coefficient α = 0.78 in the current study. Appendix A shows items of the scale. Based on items of this scale, we operationally define PWSH as the extent to which the organization supports healthy living and champions health campaigns. PWSH is a measure of healthy habits in an organization and the presence of health champions who support employees to maintain these habits and health. Some of the items of the scale are “overall, my workplace supports me in living a healthier life” and “most employees here have healthy habits”.

Further to the above, we operationally define continuous work-related sitting time as the longest uninterrupted time spent sitting while travelling to or from work and while at work. This was measured by asking participants to report the longest uninterrupted time (in minutes) spent sitting on a typical day while (1) travelling as a driver or passenger (e.g., public transportation, including travelling with a taxi) to work; (2) travelling as a driver or passenger back from work; (3) working around a desk with or without a screen; (4) in a meeting at work, and (5) eating or chatting with friends during lunchtime and when doing other things. We summed up data on the five domains to generate a composite measure of continuous work-related sitting time [i.e., sum of continuous sitting time]. Appendix B shows more information on how the five domains of continuous work-related sitting time were measured and operationalised. As this appendix indicates, work-related sitting time on a typical weekday in the previous week [last 7 days] were reported.

Other variables measured were personal and job characteristics (e.g., gender, education, physical function, marital status, chronic disease status, and income) reported in the literature as potential correlates of work-related sitting [6]. These variables were measured as potential confounding variables. Appendix B shows how these characteristics were measured, coded, and operationalized. Physical function was the extent to which the individual could perform physical tasks (e.g., walking, lifting objects) unaided. It was measured with a single item accompanying four descriptive anchors (See Appendix B). All categorical variables were dummy coded for regression analysis.

The questionnaire used had three sections; the first section presented questions measuring the personal and job characteristics whereas the second and third sections measured work-related sitting time and PWSH respectively. We tried to avoid or minimise common methods bias associated with the cross-sectional design by following procedures recently used [24]. Firstly, we presented scales in unique blocks of information, ensuring that the participants responded based on the right context [24, 25]. The second step was Harman’s one-factor method in which exploratory factor analysis with varimax rotation was used to assess the factor structure of the psychometric scale used to measure PWSH [24, 25]. The factor solution reached included two factors (factor 1 = 34.2%, and factor 2 = 21.2%; factor loadings ≥ 0.5), with the first factor accounting for a variance of less than 40% as recommended [25]. Thus, common methods bias was not an issue.

### Data collection

This study received ethics review and approval from Africa Centre for Epidemiology’s ethics review board with ethics review number 02-2021-ACE. All the participants provided a written informed consent. Questionnaires in sealed and stamped envelopes were delivered to participants at their workplaces through two private courier drivers, each supported by a research assistant. Participants were asked to complete and return questionnaires through the courier drivers over two weeks. Those who could not complete the questionnaire within the first two weeks were given an extra week to return their completed questionnaires. Research assistants called participants each week to remind them to complete the survey. Data collection was completed after four weeks (June 7 to July 20, 2021). One thousand and fifty-six (1056) questionnaires out of 1296 administered were returned, but 56 of them could not be analysed because 21 were not completed at all whereas 35 were partly completed. Thus, 1000 questionnaires were analysed.

### Statistical analysis method

Data were analysed in three main stages with IBM SPSS version 28. The first stage of the analysis was an exploratory analysis aimed at testing assumptions governing the use of the independent samples *t*-test and hierarchical linear regression analysis, which were both used to present the results. To identify missing items, we summarised all variables with descriptive statistics [i.e., frequency, per cent], enabling us to visualize the proportions of missing data associated with each variable. Nine (9) of the variables [i.e., categorical variables = 5; continuous variables = 4] had missing data. In harmony with previous studies [24, 26], we proceeded to analyse the data with these missing data through multiple inputation because data were missing at random. We plotted stem-and-leaf plots on all dependent variables [i.e., sum of continuous sitting time and its five domains] to identify outliers. These plots did not find any outliers. Following previous studies [24, 27], we further assessed and confirmed relevant assumptions [e.g., normality of the data, homogeneity of variances] for using the chosen statistical tools. Appendix C shows all the assumptions evaluated [for t-test and regression], procedures followed, and decisions reached. The final aspect of the exploratory analyses was a sensitivity analysis used in previous studies [24, 26] to identify the ultimate confounding variables for regression analysis. In this regard, the ultimate confounders are variables likely to confound the primary relationships of interest and therefore affect the regression models. Adjusting for the ultimate confounders instead of the potential confounders is recommended when multiple confounders are involved in an analysis. The sensitivity analysis is a way to remove irrelevant confounders from the actual statistical analysis [26]. Appendix D shows the various steps taken to perform this analysis.

The second phase of the analysis was intended to address the first research question. To start, we computed descriptive statistics [i.e., the mean and standard deviation] of the overall work-related sitting time [i.e., sum of continuous sitting time] and its five domains. Following this, we employed the independent samples *t*-test to compare the averages computed between groups accompanied by the three categorical variables [i.e., industry, sector, type of job]. To analyse the second research question, we first computed bivariate correlations between all measures of sitting time, PWSH, and the ultimate confounder [i.e., physical function] retained in the sensitivity analysis. We subsequently fitted 12 regression models: 6 baseline models and 6 ultimate models. The first 6 baseline models [i.e., models 1–6] assessed the associations between all measures of sitting time and PWSH. The remaining 6 models (i.e., 7–12) modified upon models 1–6 by only incorporating physical function. As such, the conclusions of this study are based on the ultimate models. Findings from the baseline and ultimate models are compared to understand the impact of the confounder on regression weights in the ultimate models. The statistical significance of the results was detected at a minimum of *p* < 0.05.

## Results

Table 1 shows summary statistics on participants’ characteristics and the main variables of the study. About 50% (*n* = 500) of the participants were men whereas the average age of participants was about 34 years (Mean = 33.97; SD = 8.7). ‘Sum of continuous sitting time’ was about 172 min (Mean = 172.15; SD = 17.67). Table 2 shows a summary of the results from the sensitivity analysis. As the table indicates, only physical function was retained as the ultimate confounder as it accounts for more than 10% of a change in the primary regression weight between PWSH and sum of continuous sitting time.

**Table 1:** Summary statistics on variables of the study

Variable type	Variable	Group	Frequency/Mean	Percent (%)/SD
Categorical variables	Gender	Men	500	50
		Women	490	49
		Missing	10	1
		Total	1000	100
	Industry	Manufacturing	100	10
		Service	870	87
		Missing	30	3
		Total	1000	100
	Sector	Public	870	87
		Private	120	12
		Missing	10	1
		Total	1000	100
	Job type	Full-time	800	80
		Part-time	180	18
		Missing	20	2
		Total	1000	100
	Chronic disease status	None	810	81
		≥ 1	170	17
		Missing	20	2
		Total	1000	100
	Marital status	Not married	300	30
		Married	660	66
		Missing	40	4
		Total	1000	100
Continuous variables	Physical function	---	2.78	0.18
	Income ($\mbox{\textcent}$)	---	1246.86	745.67
	Education (yrs)	---	18.23	4.12
	Job tenure (yrs)	---	5.29	4.32
	Age (yrs)	---	33.97	8.70
	Sitting time_to work (mins/day)	---	39.31	31.72
	Sitting time_return (mins/day)	---	40.62	38.19
	Sitting with a desk (mins/day)	---	38.31	34.16
	Sitting time_meetings (mins/day)	---	35.03	13.10
	Lunchtime sitting time (mins/day)	---	21.22	11.49
	Sum of continuous sitting time (mins/day)	---	172.15	17.67

Note: --- Not applicable; SD – standard deviation; Frequency and percent apply to categorical variables whereas the mean and SD apply to the continuous variables; ‘sum of continuous sitting time’ is the sum of all continuous sitting times in all the work domains; the minimum and maximum values for physical function are 1 and 4 respectively

**Table 2:** The ultimate confounding variables identified from the sensitivity analysis with a change in beta of at least 10%

Predictor	Stage 1			Stage 2		
	β	t	*p*	Adjusted β	Change in β	% Change in β
PWSH^d^	-0.175	-5.62	< 0.001	---	---	----
Gender (ref – men)^a^	0.010	0.264	0.791	---	---	----
Education (yrs)	0.444	0.321	0.543	---	---	---
Physical function^c^	0.049	1.218	0.224	-0.197	-0.022	13%
Industry (ref – manufacturing)^b^	0.100	1.182	0.237	-0.178	-0.003	2%
Sector (ref – public)^a^	0.069	0.804	0.422	---	---	----
Job type (ref – part-time) ^b^	-0.150	-3.676	< 0.001	-0.176	-0.001	1%
Income ($\mbox{\textcent}$)^b^	0.112	2.137	0.033	-0.178	-0.003	2%
CDS (ref – ≥1) ^b^	-0.156	-3.026	0.003	-0.177	-0.002	1%
Marital status (ref – married) ^b^	0.172	4.096	< 0.001	-0.164	0.011	-6%
Job tenure (yrs)^a^	0.079	1.145	0.253	---	---	----
Age (yrs) ^b^	-0.255	-3.427	< 0.001	-0.174	0.001	-1%

Note: --- Not applicable; ^a^variables removed in the first stage of the analysis; ^b^variables removed in the second stage of the analysis; ^c^variable retained as ultimate confounder; ^d^predictor of sedentary time; CDS – chronic disease status

Table 3 shows results from the independent samples *t*-test. Regarding ‘lunchtime sitting time’, employees working in service organizations (Mean = 21.88) reported a mean score larger than employees working in manufacturing organizations (Mean = 17.5). Employees working in private organizations reported a higher ‘sitting time_return’ (Mean = 52.25) compared with employees from public organizations (Mean = 39.29). Full-time employees reported longer ‘sitting time_to work’ and ‘sitting time_return’ compared with part-time employees. Full-time employees also reported higher ‘sum of continuous sitting time’ (Mean = 180.38) compared with part-time employees (Mean = 141.67).

Table 4 shows the correlation between relevant variables. It can be seen that ‘sum of continuous sitting time’ and its domains [except ‘sitting with a desk’] are negatively correlated with PWSH at *p* < 0.001. For example, there is a negative correlation between ‘sum of continuous sitting time’ and ‘sitting time_to work’ [*r* = -0.154; *p* < 0.001; two-tailed]. Table 5 shows the results of the hierarchical linear regression analysis. As models 1–6 [i.e., the baseline models] suggest, only ‘sitting with a desk’ is not associated with PWSH. The ultimate models [i.e., models 7–12] similarly suggest that PWSH is negatively associated with ‘sum of continuous sitting time’ [β = -0.2; t = -6.08; *p* < 0.001] and its domains, except for ‘sitting with a desk’. More specifically, lower sum of continuous sitting time is associated with higher scores of PWSH. All the models, except models 3 and 9, produce a significant F-test at *p* < 0.001. The Durbin-Watson and *tolerance* statistics of all models were also satisfactory.

**Table 3:** Bivariate correlations between relevant variables

Variable	1	2	3	4	5	6	7	8
1. PWSH	1	− 0.154**	− 0.121**	0.000	− 0.254**	− 0.174**	− 0.175**	0.155**
2. Sitting time_to work (mins/day)		1	0.789**	0.302**	0.352**	0.390**	0.797**	0.009
3. Sitting time_return (mins/day)			1	0.165**	0.303**	0.447**	0.764**	0.032
4. Sitting with a desk (mins/day)				1	0.422**	0.302**	0.605**	0.053
5. Sitting time_meetings (mins/day)					1	0.566**	0.737**	-0.013
6. Lunchtime sitting time (mins/day)						1	0.697**	0.081*
7. Sum of continuous sitting time (mins/day)							1	0.035
8. Physical function								1

**p < 0.001; *p < 0.05; PWSH – perceived workplace support for health; ‘sum of continuous sitting time’ is the sum of all continuous sitting times in all the work domains

**Table 4:** A *t*-test comparison of group means across industry, sector, and job type

Variable	Industry			Sector			Job type		
	Group	*n*	Mean	Group	*n*	Mean	Group	*n*	Mean
Sitting time_to work (mins/day)	Manufacturing	100	40.2	Public	860	39.65	Full-time	800	41.84**
	Service	860	39.88	Private	120	38.5	Part-time	170	29.41**
Sitting time_return (mins/day)	Manufacturing	100	44.7	Public	860	39.29**	Full-time	800	43.70**
	Service	860	40.74	Private	120	52.25**	Part-time	170	28.53**
Sitting with a desk (mins/day)	Manufacturing	90	42.78	Public	860	38.01	Full-time	790	38.72
	Service	860	38.36	Private	110	42.27	Part-time	170	37.35
Sitting time_meetings (mins/day)	Manufacturing	100	32.5	Public	860	35.56	Full-time	800	35.48
	Service	860	35.44	Private	120	32.92	Part-time	170	33.82
Lunchtime sitting time (mins/day)	Manufacturing	100	17.50**	Public	850	21.47	Full-time	790	21.39
	Service	850	21.88**	Private	120	19.58	Part-time	170	20.88
Sum of continuous sitting time (mins/day)	Manufacturing	100	173.4	Public	870	171.74	Full-time	800	180.38**
	Service	870	174.03	Private	120	182	Part-time	180	141.67**

**p < 0.001; all significant differences were at ‘equal variances not assumed’; the sample for each variable is less than 1,000 owing to missing data; pairs with significant differences are in **bold**; ‘sum of continuous sitting time’ is the sum of all continuous sitting times in all the work domains

**Table 5:** The associations between work-related sitting time, physical function, and PWSH

Model	Dependent variable	Predictor	Coefficients			95% CI	Model fit			
			B	SE	β(t)		R^2^	Adjused R^2^	Durbin Watson	F
1	Sitting time_to work (mins/day)	(Constant)	66.2	5.59	(11.85)**	± 21.94	0.024	0.023	---	23.91**
		PWSH	-1.74	0.36	-0.15(-4.89)**	± 1.40				
2	Sitting time_return (mins/day)	(Constant)	66.16	6.76	(9.79)**	± 26.53	0.015	0.014	---	14.75**
		PWSH	-1.65	0.43	-0.12(-3.84)**	± 1.69				
3	Sitting with a desk (mins/day)	(Constant)	38.25	6.11	(6.26)**	± 23.97	0.00	-0.00	---	0.000
		PWSH	0.00	0.39	0.00(0.01)	± 1.52				
4	Sitting_meetings (mins/day)	(Constant)	86.98	6.4	(13.59)**	± 25.12	0.064	0.063	---	68.08**
		PWSH	-3.36	0.41	-0.25(-8.25)**	± 1.60				
5	Lunchtime sitting (mins/day)	(Constant)	41.6	3.75	(11.09)**	± 14.72	0.030	0.029	---	30.52**
		PWSH	-1.33	0.24	-0.17(-5.52)**	± 0.94				
6	Sum of continuous sitting time (mins/day)	(Constant)	285.29	20.46	(13.94)**	± 80.31	0.031	0.03	---	31.59**
		PWSH	-7.34	1.31	-0.18(-5.62)**	± 5.12				
7	Sitting time_to work (mins/day)	(Constant)	63.8	7.62	(8.37)**	± 29.91	0.029	0.027	1.68	13.71**
		PWSH	-1.96	0.38	-0.17(-5.23)**	± 1.47				
		Physical function	6.69	6.03	0.04(1.11)	± 23.68				
8	Sitting time_return (mins/day)	(Constant)	60.09	9.18	(6.55)**	± 36.03	0.020	0.018	1.89	9.64**
		PWSH	-1.93	0.45	-0.14(-4.28)**	± 1.77				
		Physical function	12.06	7.27	0.06(0.10)	± 28.53				
9	Sitting with a desk (mins/day)	(Constant)	30.71	8.24	(3.73)**	± 32.32	0.003	0.002	1.60	1.40
		PWSH	-0.18	0.41	-0.02(-0.45)	± 1.59				
		Physical function	10.83	6.52	0.06(1.66)	± 25.59				
10	Sitting_meetings (mins/day)	(Constant)	84.61	8.7	(9.72)**	± 34.15	0.070	0.068	1.71	34.7**
		PWSH	-3.56	0.43	-0.27(-8.32)**	± 1.68				
		Physical function	6.35	6.89	0.03(0.92)	± 27.04				
11	Lunchtime sitting (mins/day)	(Constant)	31.14	5.1	(6.11)**	± 20.00	0.044	0.042	1.89	21.05**
		PWSH	-1.5	0.25	-0.20(-5.99)**	± 0.98				
		Physical function	13.66	4.04	0.11(3.38)**	± 15.85				
12	Sum of continuous sitting time (mins/day)	(Constant)	259.35	27.83	(9.32)**	± 109.24	0.039	0.037	1.67	19.10**
		PWSH	-8.28	1.36	-0.20(-6.08)**	± 5.34				
		Physical function	44.61	22.1	0.07(2.02)*	± 86.74				

**p < 0.001; *p < 0.05; ---Not applicable; Models 1–6 are the baseline models whereas models 7–12 are the adjusted (ultimate) models; PWSH – perceived workplace support for health; SE – standard error (of B); CI – confidence interval (of B); tolerance ≥ 0.2 for each predictor of models 7–12; ‘sum of continuous sitting time’ is the sum of all continuous sitting times in all the work domains

## Discussion

This study compared continuous work-related sitting time and its domains between full-time and part-time workers, private and public sector workers, and workers in the manufacturing and services sectors. The associations between continuous work-related sitting time and its domains as well as PWSH were also assessed.

Employees working in service organizations reported longer sitting time during lunch compared with employees working in manufacturing organizations. Researchers [18, 21] have revealed that employees in service organizations, compared with those in manufacturing firms, spend more time sitting. Jobs in service organizations require sitting for most of the working time. Consequently, sitting may become a traditional habit among employees of service organizations. If so, employees in this sector would report longer time sitting during lunch. More so, employees of manufacturing organizations sit less at work as they spend a significant part of their time doing manual labour and other forms of physical activity [18, 21]. Even so, employees in manufacturing firms may spend more time sitting at lunch since they may have worked on their feet earlier in the day; lunch time would offer these employees an opportunity to have as much rest as possible through sitting.

This study further found that employees working in private firms reported longer sitting time for travelling back home compared with employees from public organizations. Employees in the private sector in Ghana earn higher pay and receive reward packages (e.g., car loans) that are not available to most employees in public organizations [28–30]. Possibly, therefore, a larger number of employees in the private sector drove back home in their private vehicles and, therefore, spent more time sitting while waiting in traffic. The average worker in a public organization in Ghana travels to and from work in a taxi or public transport across multiple transits. Bouts of sitting while traveling from or to work among such employees would be shortened by multiple transits. This means that continuous sitting while travelling from or to work is less likely among public sector employees. We would also want to mention that our work-related sitting times are smaller than times reported in most previous studies [6, 12] because this study measured the longest work-related sitting bout on a typical day, rather than total time from all episodes of sitting at work per day.

Furthermore, this study found that full-time employees, compared with part-time employees, reported longer continuous sitting time . Similarly, full-time employees reported longer sitting time for travelling to work and returning home compared with part-time employees. These findings are consistent with the study of De Cocker et al. [2014] that reports higher work-related sitting time in Australia for full-time employees, compared with part-time employees. To explain, part-time employees spend less than the maximum time spent by full-time employees at work. So, part-time employees would be less exposed to work-related sitting in a any organization. The above group differences in sitting time suggest that some employee categories such as individuals working full-time and in service organizations face higher occupational sitting risks. This being the case, basic workplace health promotion programmes may prioritise these workgroups. Moreover, the need for these programmes in service organizations requiring more work-related sitting time may be higher.

This study found that higher PWSH was associated with shorter sitting time, though our measure of work-related sitting time does not include sedentary behaviour items. This result suggests that workplace support for health programmes not specifically designed to discourage sedentary behaviour can be associated with shorter sitting time among employees. Apart from sitting with a desk, all domains of work-related sitting time were negatively associated with PWSH. Thus, PWSH may inform or encourage employees to avoid excessing sitting. This result is analogous to findings from previous studies. A randomised controlled trial confirmed a reduced occupational sitting time linked to a workplace intervention. Employees in a qualitative study [31] reported benefits from workplace health promotion interventions. In contrast, a systematic review [5] found that workplace interventions aimed at reducing sitting time did not significantly reduce employees’ work-related sitting. So, though there are mixed findings in the literature, this study and empirical evidence to date show that workplace support for health and specialized workplace interventions can be associated with lower sitting time. The mixed findings available in the literature may be due to variations in study design. Apart from focusing on continuous work-related sitting time, the current study employed a cross-sectional design and a subjective measure of basic PWSH whereas experimental and quasi-experimental designs assessing more specialised workplace health promotion programmes were predominantly employed in the previous studies [5].

The confirmed association between PWSH and work-related sitting time was identified through between-person analysis rather than within-person analysis. This point means that while employees who perceived higher PWSH would report a shorter occupational sitting time as a group, an individual with the same perception may report longer sitting time. Thus, a negative relationship between sitting time and PWSH is a product of between-person analysis.

### Implications for policy and research

The foregoing negative relationship also supports some explanations of the Health Belief Model [HBM] and the salutogenic model, which have both been used to justify workplace health promotion programmes. Recent reviews of the HBM [16, 32] indicate that workplace support for health is a form of health promotion or education that improves employees’ health literacy, and therefore, enables them to modify behaviour such as avoiding excessive sitting while working. Employees are influenced by these programmes to modify their behaviour because they value health and health-seeking behaviours after participating in these programmes. Similarly, workplace interventions for health are founded on the concept of salutogenesis, which proposes a focus on interventions preventing disease (e.g., programmes reducing sitting time) rather than on factors that cause disease [17, 32].

Deductively, instituting workplace support for health programmes is a way to implement propositions from the HBM and the salutogenic model. Our results support a need for the implementation of basic workplace health promotion programmes, which should be based on the availability of policies for PWSH, health education activities for employees, and health campaigns from health champions [15]. As indicated earlier, a basic programme of this type should be the foundation of more advanced or specific interventions.

### Limitations

We admit that this study has some limitations that future researchers and decision-makers should consider. First, participants were selected with a non-probability sampling method, so our findings may not be generalised to the general population of employees. Our unequal samples from services and manufacturing firms may also limit the generalizability of our findings. Future studies utilizing representative national or regional samples are encouraged. Our use of subjective measures (of work-related sitting time), instead of device-based measures (e.g., digital timers of sitting) is also considered a limitation as this method was subject to recall bias. We tried to avoid or minimise this limitation by asking participants to report sitting times from the previous week rather than from the last three months [6]. Future researchers can avoid this limitation by using device-based measures (e.g., physical activity trackers) through cross-sectional, longitudinal, or randomised controlled studies.

As a cross-sectional design, this study could not have eliminated or adjusted for all confounding of the relationships assessed. Physical activity outside work, health risk perception, the individual’s job role in the organization, and lifestyle factors were not considered in this study, although they could confound the relationships examined. Future researchers may consider these variables as potential covariates. A variable such as the distance between employees’ residence and their places of work could not be measured, although this could have confounded the primary relationships. Future researchers can use GIS [Geographic Information System] to measure this variable. Though this study was conducted after a Coronavirus disease 2019 (COVID-19) lockdown in Ghana was lifted, social distancing measures observed by individuals and organizations may have affected reported sitting times. Findings from this study such as the amount of sitting time reported may differ from findings reported by previous researchers [4, 8]. Specifically, the sum of continuous sitting time would be smaller than what previous studies [4, 8] have reported because total sitting time is the sum of all episodes of sitting in the day whereas our study focused on only the longest episode in each work domain. This difference may have affected the associations tested in this study. For these reasons, future researchers should exercise caution in comparing our results to results based on other measures of total sitting time. Our comparison of sitting time between groups (e.g., full- and part-time workers) has yielded some useful results, but our analysis suggests a need for other groups to be considered in the future. Retired employees, for instance, may report longer sitting times depending on their current occupation.

### Strengths

Despite the above limitations, this study is important for several reasons. First, it is novel for being the first to assess some understudied domains of work-related sitting time. More so, this study is the first to assess the associations between work-related sitting time and PWSH. If so, this study is an important antecedent for future studies and provides a foundation for a potential randomized controlled trial. For example, statistics [e.g., standardised regression coefficients] from this study can be used to compute future sample sizes. This study also adopted a robust analysis including a sensitivity analysis minimising confounding bias and recommended procedures against common methods bias. Comparing the baseline and ultimate models was an extension of our sensitivity analyses that enabled us to identify the importance of adjusting for confounding variables in this study. Crude regression weights from the baseline models are different from those in the ultimate models, suggesting that the ultimate confounder had some influence on the primary relationships tested. These methodological qualities of the study are cognisant of checklist items from STROBE [Strengthening the Reporting of Observational Studies in Epidemiology] [24, 33]. Appendix E shows items of STROBE met. These methods are replicable and can be used by future researchers to strengthen their cross-sectional designs.

## Conclusion

Employees working in service firms, compared with those working in manufacturing firms, reported longer sitting times for lunchtime whereas employees of private organizations, compared with those from public organizations, reported longer sitting times for travelling back home in a car. Full-time employees, compared with part-time employees, reported longer sitting times for travelling to and from work in a car. Sum of continuous sitting time is lower at higher PWSH. PWSH is associated with shorter sitting time for four domains (except for ‘sitting with a desk’). Sitting around a desk is a primary job task that may be difficult to modify or avoid. Yet, it can cause shoulder, neck, and low-back pain among employees. Given our result, interventions such as PWSH may be unable to modify sitting around a desk, but future research is needed to substantiate our evidence. It is concluded that continuous sitting time is associated with employees’ job type, as well as industry and sector of employment. Interventions providing support for health at work can discourage continuous work-related sitting, except sitting around a desk. This study reinforces the importance of workplace support for health and its role in work-related sitting.

## Electronic supplementary material

Below is the link to the electronic supplementary material.

Appendix A. The Workplace Support for Health Scale.

Appendix B. Operationalization of variables.

Appendix C. Statistical assessment of relevant assumptions.

Appendix D. steps taken at the two stages of the sensitivity analyses.

Appendix E. STROBE Statement—checklist of items that should be included in reports of observational studies.

Supplementary Material 6

## Data Availability

Data used for this study are available in the name DATA FILE as an online supplementary material.
