# Supplementary material for: Continuous work-related sitting time and its association with perceived workplace support for health among workers in the Greater Accra Municipality: a cross-sectional analysis with sensitivity analyses
Source: BMC Public Health. 2024 Nov 5;24:3057. doi: 10.1186/s12889-024-20572-z (PMC11539606; doi:10.1186/s12889-024-20572-z)
Supplement: Supplementary file 1 — Appendix A. The Workplace Support for Health Scale. [file 12889_2024_20572_MOESM1_ESM.doc]

**Appendix A. The Workplace Support for Health Scale**

The following statements measure workplace support for health at your organization**.** On a scale of 1 to 5, where **1 – strongly disagree, 2 – disagree, 3 – somewhat agree, 4 – agree, and 5 – strongly agree**, indicate the extent to which you agree or disagree with the following statements about your organization.

| # | Statement | 1 | 2 | 3 | 4 | 5 |
| --- | --- | --- | --- | --- | --- | --- |
| 1 | Overall, my workplace supports me in living a healthier life. |  |  |  |  |  |
| 2 | My supervisor supports me in living a healthier life. |  |  |  |  |  |
| 3 | Most employees here have healthy habits. |  |  |  |  |  |
| 4 | At my workplace, we have one or more leaders (e.g., CEOs or managers) who are wellness champions. |  |  |  |  |  |
| 5 | At my workplace, we have one or more employees who are wellness champions. |  |  |  |  |  |

Note: CEOs – Chief executive officers
