# Supplementary material for: Continuous work-related sitting time and its association with perceived workplace support for health among workers in the Greater Accra Municipality: a cross-sectional analysis with sensitivity analyses
Source: BMC Public Health. 2024 Nov 5;24:3057. doi: 10.1186/s12889-024-20572-z (PMC11539606; doi:10.1186/s12889-024-20572-z)
Supplement: Supplementary file 2 — Appendix B. Operationalization of variables. [file 12889_2024_20572_MOESM2_ESM.doc]

Appendix B. Operationalization of variables

| No. | Variable | Definition | Type | Groups (codes) |
| --- | --- | --- | --- | --- |
| 1 | Gender | The sex (male or female) of the employee | Categorical | Male (1), female(2) |
| 2 | Industry | Whether the employee worked in a service or manufacturing organization | Categorical | Manufacturing (1), service (2) |
| 3 | Sector | Whether the employee worked in a public or private organization | Categorical | Public(1), private (2) |
| 4 | Job type | Whether the employee was a full-time or part-time employee | Categorical | Full-time (1), part-time (2) |
| 5 | Chronic disease status | Whether the individual had at least 1 chronic condition diagnosed by a physician | Categorical | None (1), ≥ 1(2) |
| 6 | Marital status | Whether the employee was married or not | Categorical | Not married (1), Married (2) |
| 7 | Physical funcion | The extent to which the individual can perform physical tasks (e.g., walking, lifting objects) unaided | Ordinal | Not at all (1), low extent (2), moderate extent (3), high extent (4) |
| 8 | Income (₵) | The net monthly salary of the employee in Ghana cedis | Continuous | --- |
| 9 | Education (yrs) | The employee's total number of years of schooling | Continuous | --- |
| 10 | Job tenure (yrs) | The number of years the employee had worked in the organization | Continuous | --- |
| 11 | Age (yrs) | The age (in years) of the employee | Continuous | --- |
| 12 | Sitting time_to work (mins/day) | The longest continuous amount of time (in minutes) spent by the employee sitting on a typical weekday while travelling to work in a car (e.g., private driving, public transportation, travelling with a taxi) | Continuous | --- |
| 13 | Sitting time_return (mins/day) | The longest continuous amount of time (in minutes) spent by the employee sitting on a typical weekday while travelling back home from work in a car (e.g., private driving, public transportation, travelling with a taxi) | Continuous | --- |
| 14 | Sitting with a desk (mins/day) | The longest continuous amount of time (in minutes) spent by the employee sitting on a typical weekday while working with or without a screen around a desk | Continuous | --- |
| 15 | Sitting time_meetings (mins/day) | The longest continuous amount of time (in minutes) spent by the employee sitting on a typical weekday in a job-related meeting | Continuous | --- |
| 16 | Lunchtime sitting time (mins/day) | The longest continuous amount of time (in minutes) spent by the employee sitting on a typical weekday during lunchtime (including time spent sitting while resting or doing other things) | Continuous | --- |
| 17 | Total sitting time (mins/day) | The sum of the above five domains (12-16) of work-related sitting time | Continuous | --- |

Note: --- Not applicable; all categorical variables were dummy coded; sitting times in the last 7 days (week) were reported.
