# Supplementary material for: Continuous work-related sitting time and its association with perceived workplace support for health among workers in the Greater Accra Municipality: a cross-sectional analysis with sensitivity analyses
Source: BMC Public Health. 2024 Nov 5;24:3057. doi: 10.1186/s12889-024-20572-z (PMC11539606; doi:10.1186/s12889-024-20572-z)
Supplement: Supplementary file 3 — Appendix C. Statistical assessment of relevant assumptions. [file 12889_2024_20572_MOESM3_ESM.doc]

Appendix C. Statistical assessment of relevant assumptions

| Statistical tool | Assumption | Step | Result | Decision |
| --- | --- | --- | --- | --- |
| t-test | Normality of the data (All measures of sitting time) | Stem-and-leaf plots | Satisfactory | A satisfactory stem-and-leaf plot is enough for samples as large as ours (Garson, 2012) |
|  |  | Shapiro-Wilk's test | Assumption not met at p<0.05 | For samples as large as ours, deviation from normality is not an issue (Garson, 2012) |
|  | Equality of variances | Levene's equality of variances test | Assumption not met at p<0.05 | We used the 'equal variances not assumed' option |
| Regression analysis | Normality of the dependent variables | Stem-and-leaf plots | Satisfactory | A satisfactory stem-and-leaf plot is enough for samples as large as ours (Garson, 2012) |
|  |  | Shapiro_wilk's test | Assumption not met at P<0.05 | For samples as large as ours, deviation from normality is not an issue (Garson, 2012) |
|  | Linearity | We plotted lines of best fit on the primary relationships to know if they were of a linear form | The graphs produced a satisfactory pattern recommended by Garson (2012) | Condition met for a regression analysis |
|  | Independence of errors | Durbin Watson statistics were generated through the multiple regression models | Durbin-Watson statistic falls between 1.5 and 2.4 as recommended (Garson, 2012) | Condition met for a regression analysis |
|  | Multi-collinearity | Tolerance values were computed through the multiple regression models | The tolerance values are >0.2 as recommended (Bempong and Asiamah, 2022) | Condition met for a regression analysis |
|  | Homoscedsticity | We plotted standardized residuals against standardized predicted values of the dependent variable in all regression models through which the relationships were assessed. | The graphs produced a satisfactory pattern recommended by Garson (2012) | Condition met for a regression analysis |
