# Supplementary material for: Continuous work-related sitting time and its association with perceived workplace support for health among workers in the Greater Accra Municipality: a cross-sectional analysis with sensitivity analyses
Source: BMC Public Health. 2024 Nov 5;24:3057. doi: 10.1186/s12889-024-20572-z (PMC11539606; doi:10.1186/s12889-024-20572-z)
Supplement: Supplementary file 4 — Appendix D. steps taken at the two stages of the sensitivity analyses. [file 12889_2024_20572_MOESM4_ESM.doc]

Appendix D. Steps taken at the two stages of the sensitivity analyses

| Stage | Step | Assumption |
| --- | --- | --- |
| 1 | 1 | Fit a simple linear regression model to assess the relationship between total sitting time and workplace support for health |
|  | 2 | Note the standardised regression weight from step 1 |
|  | 3 | Fit a multiple linear regression model in which all measured confounding variables are treated as predictors of the main independent variable, workplace support for health |
|  | 4 | Identify from step 3 potential confounders that have a p-value ≥0.25 |
|  | 5 | Predictors from step 4 that produced a p≥0.25 should be removed from the analysis and the others kept for the next stage of the analysis |
| 2 | 6 | Adjust for each of the remaining confounding variables in the model fitted at step 1 |
|  | 7 | Compute the per cent change between the standardised regression weight at step 1 and the new weight resulting from step 6 |
|  | 8 | All potential confounders that produce a change of 10% or more should be incorporated into the final analysis as the ultimate confounders |
